# LHCSR3 affects de-coupling and re-coupling of LHCII to PSII during state transitions in *Chlamydomonas reinhardtii*

**DOI:** 10.1038/srep43145

**Published:** 2017-02-24

**Authors:** Thomas Roach, Chae Sun Na

**Affiliations:** 1 Institute of Botany, Leopold-Franzens-Universität-Innsbruck, Sternwartestraße 15, 6020 Innsbruck, Austria; 2 Institute of Ecological Phytochemistry, Department of Plant Life and Environmental Science, Hankyong National University, Anseong 456-749, Gyeonggi-do, Republic of Korea

## Abstract

Photosynthetic organisms have to tolerate rapid changes in light intensity, which is facilitated by non-photochemical quenching (NPQ) and involves modification of energy transfer from light-harvesting complexes (LHC) to the photosystem reaction centres. NPQ includes dissipating excess light energy to heat (qE) and the reversible coupling of LHCII to photosystems (state transitions/qT), which are considered separate NPQ mechanisms. In the model alga *Chlamydomonas reinhardtii* the LHCSR3 protein has a well characterised role in qE. Here, it is shown in the *npq4* mutant, deficient in LHCSR3, that energy coupling to photosystem II (PSII) more akin to qT is also disrupted, but no major differences in LHC phosphorylation or LHC compositions were found in comparison to wild-type cells. The qT of wild-type cells possessed two kinetically distinguishable phases, with LHCSR3 participating in the more rapid (<2 min) phase. This LHCSR3-mediated qT was sensitive to physiological levels of H_2_O_2_, which accelerated qE induction, revealing a way that may help *C. reinhardtii* tolerate a sudden increase in light intensity. Overall, a clear mechanistic overlap between qE and qT is shown.

Achieving photosynthetic efficiency under naturally fluctuating light intensities requires mechanisms that can rapidly switch between highly efficient light absorption and dissipation of excess-absorbed light energy. Otherwise over-excited reaction centres lead to the elevated formation of potentially damaging reactive oxygen species (ROS) and photoinhibition^1^,^2^,^3^. Non-photochemical quenching is a collective name for the mechanisms that regulate energy transfer to the photosystem reaction centres, thereby protecting from photoinhibition.

The most rapidly inducible component of NPQ is qE, which is regulated by the pH of the thylakoid lumen. A low pH leads to protonation of a LHC-type protein triggering the switch between light harvesting and excess light-energy dissipation^4^,^5^,^6^,^7^,^8^,^9^. In higher plants this LHC-type protein is PsbS^10^, whereas in *Chlamydomonas reinhardtii* Light-Harvesting-Complex-Stress-Related-3 (LHCSR3) is involved^11^. *Arabidopsis thaliana* or *C. reinhardtii* mutants deficient in PsbS or LHCSR3, respectively, are both referred to as *npq4* and have severely diminished qE under excess light^10^,^11^. Using *npq4* and dissipaters of the trans-thylakoid pH gradient (ΔpH) it has been shown that qE protects from ROS production and photoinhibition under excess light^12^,^13^,^14^. Expression of the gene coding for LHCSR3 (previously referred to as LI818) follows diurnal cycles in day/night grown photoautotrophic cells^15^, and can be rapidly up-regulated when cells are subjected to high light under ambient CO_2_^11^, conditions that lead to excess light absorption and a need for qE.

State transitions (qT) are the reversible associations of LHCs, mainly LHCII, with PSII and PSI. Transitioning from state I, where LHCII is coupled to PSII, to state II is regulated by a thylakoid-bound kinase, which phosphorylates LHC proteins when the plastoquinone (PQ) pool becomes reduced. It is an NPQ mechanism that is much more active in algae than higher plants^16^. In *C. reinhardtii* up to 80% of LHCII de-couples energy transfer from PSII in state II, with PSI able to use 20% of this light energy^17^,^18^. STN7, the higher plant analogue of Stt7 in *C. reinhardtii*^19^, has H_2_O_2_-sensitive thiol groups^20^ that are likely conserved in Stt7, as Stt7-mediated LHC phosophoryations are decreased by H_2_O_2_^21^. Hydrogen peroxide is a ROS produced in the chloroplast under high light^22^. Reduction of cysteines 68 and 73 of Stt7 are required for Stt7 kinase activity^23^, explaining why H_2_O_2_ that promotes disulphide bridge formation inhibits LHC phosophorylation^21^. The Stt7-deficient *C. reinhardtii (stt7*) is not particularly prone to photoinhibition^24^, but the *npq4stt7–9* double mutant is more light-sensitive than *npq4*^13^. Recently, it was shown that *stt7–9* is a “leaky” mutant and still possesses residual Stt7 kinase activity^25^, including phosophorylation of LHCSR3, although with severely diminished levels of LHCII phosphorylation^13^,^21^.

Together, qT and qE act synergistically for the benefit of photosynthetic efficiency^26^. While it is clear that qE and qT each have unique mechanistic aspects, a significant component of each requires reorganisation of LHCII and could possess more structural overlap than previously recognised. After all, it has been noticed that LHCSR3 associates to PSI as part of the mobile LHC fraction during qT^13^,^25^. Here, using *npq4, stt7–9* and *stt7-7* (a non-leaky Stt7-kinase deficient mutant) it is shown that LHCSR3 is involved in a de-coupling and re-coupling of energy transfer to PSII during qT. This process was sensitive to H_2_O_2_ and, in agreement with an involvement of LHCSR3, the qT of *npq4* was much less H_2_O_2_-sensitive than wild-type cells. During high light the H_2_O_2_-sensitivity of transitioning to state I accelerated qE induction, enabling cells to adjust to high light quicker.

## Results

**Figure 1:**
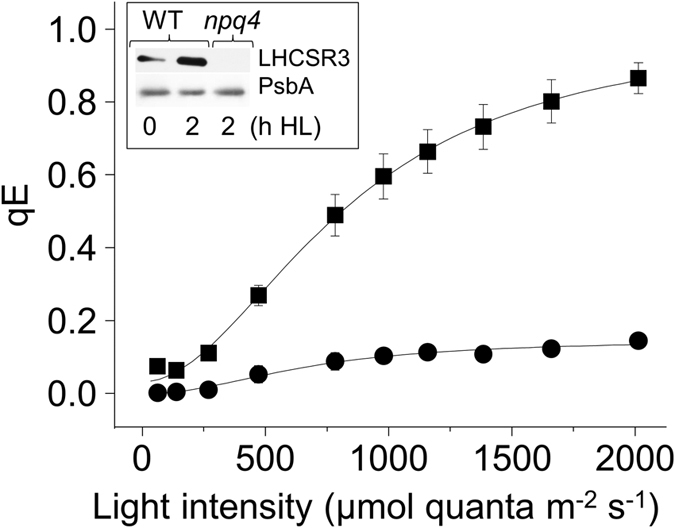
The qE phenotype of *npq4*. The NPQ parameter of qE was measured at the indicated light intensities in wild-type (squares) and *npq4* (circles). Cells were pre-treated with high light for 2 h and recovered for 1.5 h before measurements, n = 3 ± SD. The western blot inset shows the accumulation of LHCSR3 in 0 and 2 h treated wild-type cells and its absence in *npq4*. Supplementary Figure S5 shows an uncropped blot. The D1 subunit of PSII (PsbA) is shown as loading control.

**Figure 2:**
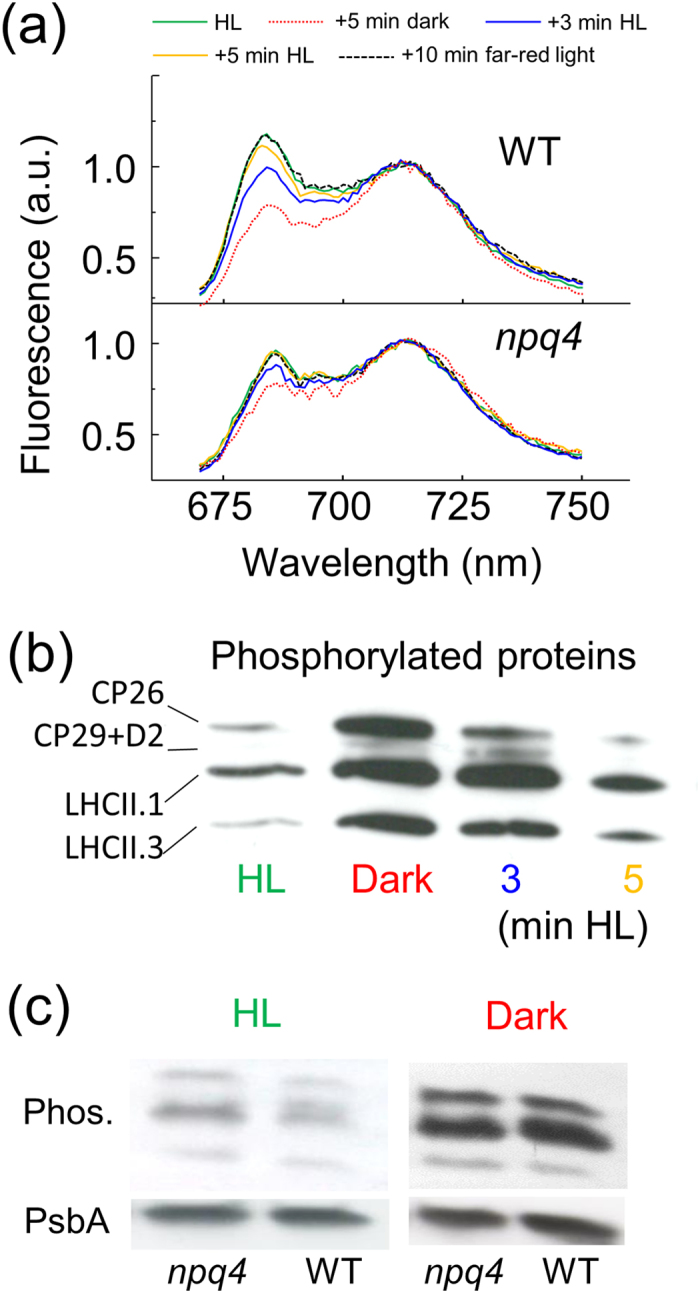
Characterisation of qT in wild-type and *npq4*. **(a)** Low-temperature (77 K) chlorophyll fluorescence emission spectra (ex: 440 nm) of wild-type in state I after 2 h high light pre-treatment (HL; green), in state II after a subsequent 5 min dark (red dotted) and then re-transitioning to state I by exposure to 3 min (blue) and 5 min (yellow) high light, and a further 10 min far-red light (black dashed). The changes in *npq4* are shown below wild-type and all spectra were normalised at 715 nm. **(b)** Modification in the level of phosphorylation of thylakoid proteins in wild-type during transitioning between states I and II. Cells were treated as in **(a)** and phosphorylated proteins were detected by immunoblotting with phospho-threonine antibody. **(c)** Levels of thylakoid protein phosphorylation (Phos.) of wild-type and *npq4* in high light (HL) and after a subsequent 4 min of darkness (Dark). Supplementary Figure S6 shows an uncropped blot. The D1 subunit of PSII (PsbA) blotted from the same membrane is shown as a loading control.

Between the light intensities of 250–2000 μmol quanta m^−2^ s^−1^ the qE of *npq4* was six times lower than in wild-type cells, a deficiency due to the absence of the light-inducible LHCSR3 protein (Fig. 1). To compare the qT of *npq4* and wild-type cells chlorophyll fluorescence at a temperature of 77 K was measured after various light treatments. Chlorophyll excitation at 77 K leads to emission peaks centred at 685 and 715 nm, corresponding to PSII and PSI, respectively, thereby indicating the location of the mobile fraction of LHCII. Dark adapting pre high light-treated wild-type cells led to a decrease of fluorescence at 685 nm, typical of a transition to state II and a de-coupling of energy transfer away from PSII, which also occurred in *npq4* (Fig. 2a). Continued respiration and ATP consumption in the dark leading to an imbalanced ATP:NADPH ratio induces NADPH reduction of the PQ pool (chloro-respiration) that activates Stt7 kinase and induction of state II^19^,^27^. Re-exposing these cells to light for 3 min, thereby re-oxidising the PQ pool, increased fluorescence at 685 nm relative to 715 nm (Fig. 2a), as expected in state I when LHCII couples energy transfer to PSII. Transitioning from state I (under high light) to state II (due to dark) and back to state I (due to light) was accompanied by a major increase then progressive decreases in LHC phosphorylation of wild-type cells (Fig. 2b), as expected^19^. Only PSBC (CP43) and PSBD (D2) proteins of the PSII complex were detectably phosphorylated by *stt7–9* in the dark (Supplementary Figure S1), as expected^25^.

In a comparison of *npq4* and wild-type cells in state I, the 77 K chlorophyll fluorescence at 685 nm (i.e. LHCII coupling with PSII) was noticeably less in *npq4* (Fig. 2a). A decrease in fluorescence from PSII could be due a higher Stt7 activity preventing cells from attaining state I. However, no major differences in LHC phosphorylation were observed between *npq4* and wild-type cells in state I or state II (Fig. 2c). Other explanations for a decreased fluorescence from PSII could be photoinhibition, smaller antenna size and higher qE, but the similar maximum quantum yield of PSII (*F*_v_/*F*_m_) after pre-high light treatment indicated limited differences in photoinhibition (Table 1), the insignificantly different maximum chlorophyll fluorescence (*F*_m_) measured in the presence of DCMU showed equal antenna size (Table 1) and qE is not higher in *npq4* (Fig. 1). Therefore, a lack of fully achieving state 1 in *npq4* could be explained by an absence of LHCSR3 restricting energy coupling to PSII. It is important to note that while traditionally a transition to state II assumed that most LHCII migrated to PSI this is now considered unlikely^18^. The definition of state II used here is that LHCII has disassociated energy transfer from PSII regardless of any association to PSI.

**Figure 3:**
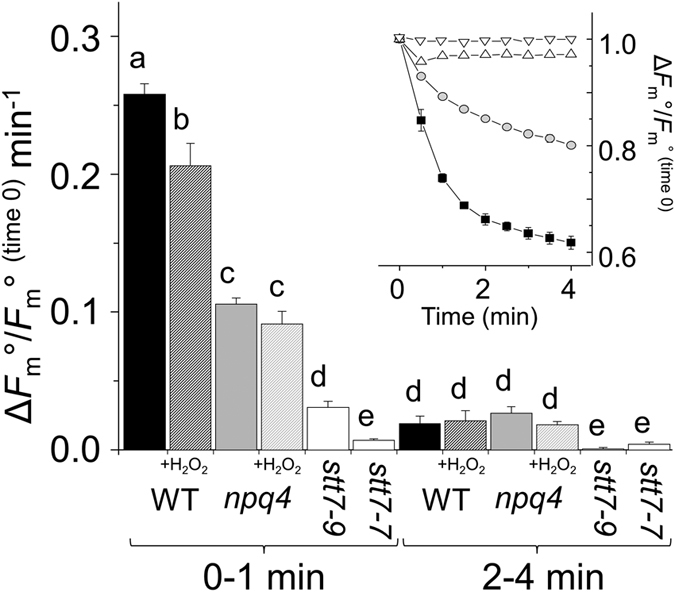
The state I to II transition of qT separated into the LHCSR3-mediated and Stt7 kinase-mediated components and effects of H_2_O_2_. High light-treated cells were subjected to far-red light to fully induce state I (*F*_m_°^FR^). State II conditions were activated by placing cells in darkness and the decrease in *F*_m_° was followed (see inset) in wild-type (WT; squares), *npq4* (circles), *stt7-7* (downward triangles) or *stt7–9* (upward triangles). The bar chart shows decrease rates in *F*_m_° of wild-type (black), *npq4* (grey) or *stt7-7*/*stt7–9* (white) during 0–1 min or 2–4 min in the absence (solid) or presence (white diagonal-stripe) of 0.1 mM H_2_O_2_ added 1 min before measurements. Data was normalised to *F*_m_°^FR^ at 0 min. Different letters indicate significant differences (*P* < 0.05), n = 4 ± SD.

At room temperature chlorophyll fluorescence is primarily from PSII. By following changes *F*_m_, qT can be measured *in vivo* by increases and decreases in *F*_m_ that correspond to transitioning to state I and state II, respectively. Cells were placed in state I with far-red light until *F*_m_ no longer increased. After switching off the far-red light and placing cells in darkness, *F*_m_ decreased as cells transitioned to state II. The greatest decrease in *F*_m_ occurred during the first minute and in *npq4* this decrease was 59% less than in wild-type (Fig. 3). Therefore, the absence of LHCSR3 affected measurements of qT in mechanism completely separate to qE (i.e. in darkness). It was observed that *npq4* has a slightly higher chlorophyll *a:b* ratio and a larger size of the xanthophyll cycle pool than wild-type cells (Table 1), indicating there are some minor differences in the LHC composition of *npq4*, as noted previously^11^, but the PSII antenna size was equal (Table 1). In *stt7–9* there was also a small, but significant decrease in *F*_m_ within the first 0.5 min after switching off the far-red light, while in *stt7-7* this decrease was even smaller. The linear decrease in *F*_m_ during 2–4 min (inset Fig. 3) was insignificantly different between *npq4* and wild-type, and can be attributed to Stt7 kinase-mediated qT by its near absence in *stt7–9* and *stt7-7*. In the presence of 0.1 mM H_2_O_2_ the initial (0–1 min) rate of *F*_m_ decrease was significantly lowered by 20% in wild-type cells, but insignificantly lowered in *npq4* (Fig. 3). H_2_O_2_ had less effect on the slower decrease in *F*_m_ (2–4 min) of wild-type and *npq4* cells.

**Figure 4:**
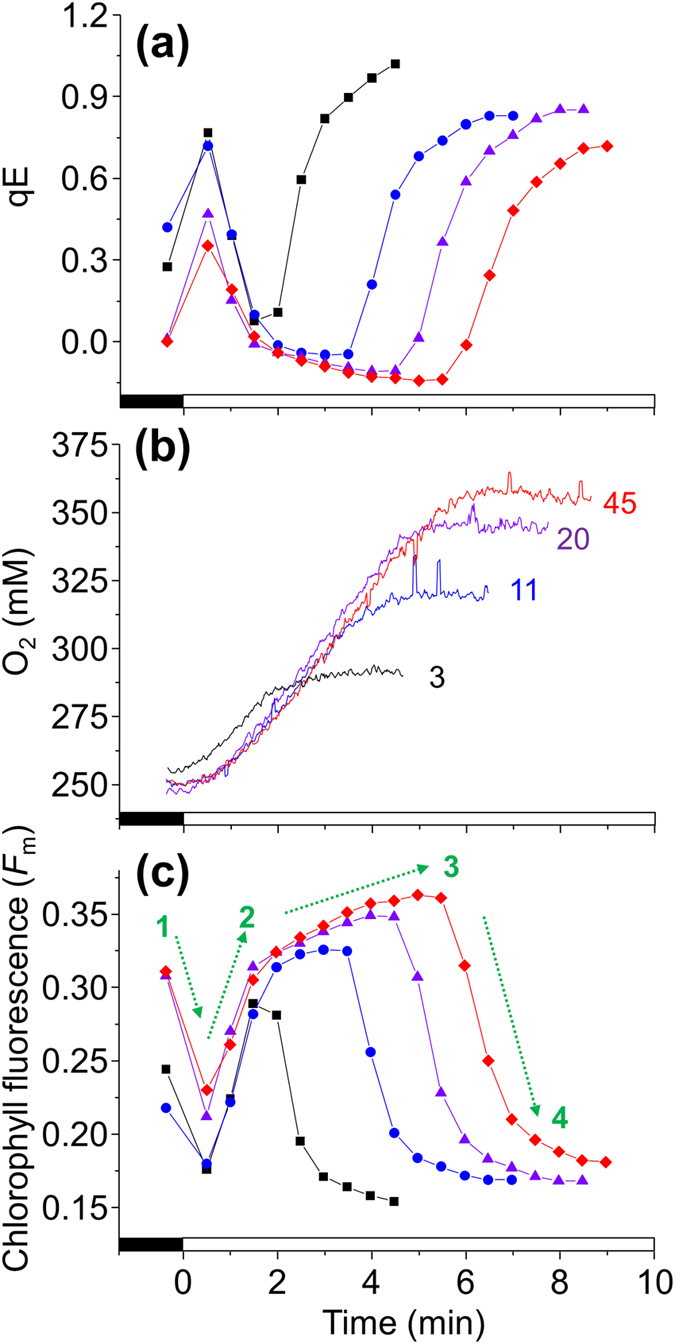
The state II to I transition of qT and qE induction during dark to light exposure. Pre high light-treated wild-type cells were placed in state II by dark-adapting for various intervals. Actinic light at 474 μmol quanta m^−2^ s^−1^, as used to induce a transition to state I and activate qE, is indicated by the white bar on the X-axis. **(a)** The qE of cells dark adapted for 3 min (squares), 11 min (circles), 20 min (triangles) or 45 min (diamonds), calculated for all using the *F*_m_^o^ after 45 min dark adaptation. **(b)** O_2_ content of the media (lines are labelled with the time of dark adaption) simultaneously measured during a fluorescence quenching analysis. **(c)** The *F*_m_’ values used to calculate qE using the same symbols in **(a)**. In **(c)** the dashed green arrows of *F*_m_ changes indicate 1) rapid qE of state II cells, 2) rapid qT transition to state I, 3) slower qT transition to state I, and 4) qE of state I cells (see text for details).

**Figure 5:**
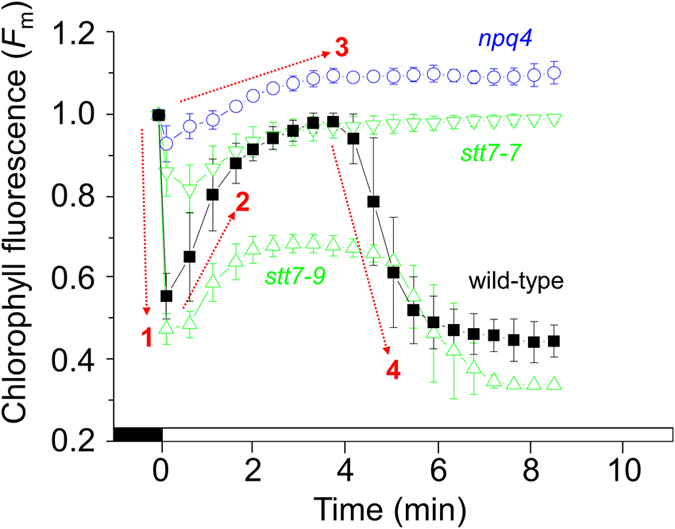
The NPQ phases during dark to light exposure separated into the LHCSR3-mediated and Stt7 kinase-mediated components. Wild-type (squares), *npq4* (circles), *stt7–9* (upward triangles) and *stt7-7* (downward triangles) were pre high light-treated and then dark-adapted for 15 min inducing state II conditions. Subsequently, cells were treated with 474 μmol quanta m^−2^ s^−1^, as indicated by the white bar on the X-axis, to induce state I before qE became induced. Data are normalised to *F*_m_° at 0 min. The dashed red arrows of *F*_m_ changes in wild-type cells indicate 1) rapid LHCSR3-dependent qE, 2) rapid LHCSR3-involved qT transition to state I, 3) slower Stt7-mediated qT transition to state I, and 4) LHCSR3- and Stt7-dependent qE of state I cells (see text for details).

Deciphering chlorophyll fluorescence for measuring qE in *C. reinhardtii* can be problematic because of the large overlap of qT. For example, qE apparently decreased to negative values during a quenching analysis because *F*_m_’ (*F*_m_ measured in the light) increased above *F*_m_° (*F*_m_ measured in the dark) (Fig. 4). This can be explained by cells transitioning from state II to state I when they are transferred from dark to light, as shown by 77 K chlorophyll fluorescence in Fig. 2a. In low light-acclimated wild-type cells, which only accumulated low levels of LHCSR3 (Fig. 1), there was an absence of the rapid light-induced rise in *F*_m_’ (Supplementary Figure S2). Simultaneously measuring net O_2_ production during the quenching analysis helped separate the qE and qT responses. High light treated cells were dark-adapted to allow qE to relax and to place them in state II. Subsequently, during the quenching analysis, despite differences in the time before qE was induced (Fig. 4a) it only occurred when net O_2_ production ceased (Fig. 4b). Most likely, qE became induced once CO_2_ became depleted^21^. Longer recovery times permitted more CO_2_ to dissolve into the media that became CO_2_-depleted by photosynthesis during the high light pre-treatment. Dark-adapting wild-type cells for only 3 min led to qE being induced before cells fully transitioned to state I, whereas 20–45 min dark adaption enabled sufficient recovery time to reveal two qT kinetics: First, an initial rapid increase in *F*_m_’ between 0.5–2 min of illumination that partially occurred in *stt7–9* and to a lesser extent in *stt7-7*, but was absent in *npq4*, and a second slower increase that was absent in *stt7–9* and *stt7-7*, but present in *npq4* (Figs 4c and 5). To summarise, with the help of the arrows in the quenching analysis shown in Figs 4c and 5, the initial NPQ phases of wild-type cells can be assigned in order of their occurrence to an immediate LHCSR3-dependent qE followed by an LHCSR3-dependent rapid qT transition to state I that overlaps with the slower Stt7-mediated qT transition to state I.

**Figure 6:**
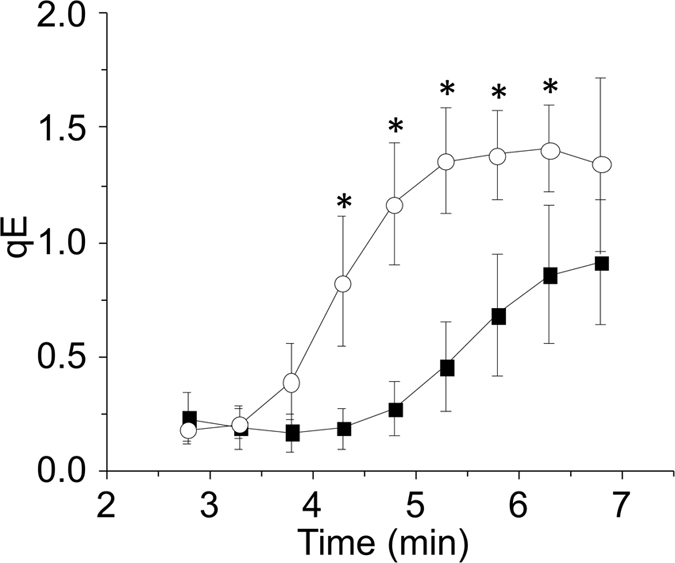
H_2_O_2_ accelerates the induction of qE. Wild-type cells were pre high light-treated and then dark-adapted for 15 min in the presence (closed squares) or absence (open circles) of 500 U mL^−1^ catalase. Cells were subsequently treated with 474 μmol quanta m^−2^ s^−1^ to induce qE. Significant differences (*P* < 0.05) in the presence or absence of catalase at each interval are indicated by *.

With further light treatment qE became induced, as shown by the decrease in *F*_m_’ that could be prevented by the ΔpH-dissipater nigericin (Supplementary Figure S3). Despite that *stt7-7* had no less LHCSR3 than *stt7–9* (Supplementary Figure S4), *F*_m_’ of *stt7-7* did not decrease by the time qE had been induced in wild-type or *stt7–9* (Fig. 5). Therefore, although qE was LHCSR3 dependent (i.e. absent in *npq4*) there was further control mediated by Stt7 only apparent in the non-leaky *stt7-7*. When the quenching analysis of wild-type cells was made in the presence of catalase the light-induced transition to state I was delayed^21^, as was the induction of qE (Fig. 6). The same slowing of qE induction from the addition of catalase was also observed with *stt7–9*, but not *stt7-7* that was unable to induce qE during the analysis (Supplementary Figure S4).

## Discussion

The NPQ mechanisms of qE and qT have each been characterised under distinct conditions, leading to the notion of completely unique processes. For example, qT has been investigated in conditions such as anoxia in the dark^17^,^18^,^19^,^27^, which is far away from the excess light required for inducing qE. However, Tokutsu and Minagawa^5^ showed that the majority of LHCSR3 in high light-treated *C. reinhardtii* was associated with detached LHCII, a situation that could have derived from either qE or qT. Moreover, others have shown LHCSR3 attached to the PSI-supercomplex^13^,^25^, fitting with a role for LHCSR3 in qT. As discussed below, our data would fully support a role for LHCSR3 in energy coupling of LHCII to PSII as part of qT.

Placing *C. reinhardtii* from high-light or far-red light into darkness activates chlororespiration, a reduction of the PQ pool and transition to state II^13^,^27^. Two kinetically separate phases were evident in wild-type cells during this transition, with *npq4* retarded in the initial rapid decrease of *F*_m_° (Fig. 3). However, the later linear and slower decrease of *F*_m_° from 2–4 min, absent in *stt7* mutants, was equally present in *npq4* and wild-type alongside equally phosphorylated LHCs after 4 min dark in *npq4* and wild-type (Fig. 2c). In summary, *npq4* was inhibited in the rapid de-coupling of energy to PSII during a transition to state II, revealing the involvement of LHCSR3, but *npq4* was not affected in the slower de-coupling of energy that was attributable to Stt7 kinase.

A role for LHCSR3 in energy coupling to PSII during transition to state I was also explored. Exposing dark-treated cells in state II to actinic light induces transition to state I^13^, as shown by changes in chlorophyll fluorescence at 77 K (Fig. 2a). A light-induced transition to state I can be called the “S” (semi-steady state) to “M” (maximum) rise when using the so-called O-J-I-P-S-M nomenclature^28^. Here, it was observed during a quenching analysis that the increase in *F*_m_’ of wild-type cells possessed two kinetically separate phases; a rapid initial increase partially present in *stt7* mutants, but totally absent in *npq4* (Fig. 5), and a second slower increase over several minutes (Fig. 4c) that occurred alongside a decrease in LHC phosphorylation (Fig. 2b), explaining its absence in *stt7* mutants (Fig. 5). A lack in the rapid *F*_m_’ increase early in the quenching analysis by *npq4* confirms an involvement of LHCSR3, and also explains why a high light pre-treatment to induce LHCSR3 was required to see this phenomenon (Supplementary Figure S2). This also explains why the rapid increase in *F*_m_’ was also observed in *stt7–9*, and to a lesser extent in *stt7–7* (Fig. 5). It is known that *stt7–9* is a leaky mutant with residual Stt7 activity^25^. However, measuring qT by shifting far-red light-treated cells to dark showed *stt7-7* and *stt7–9* behaved very similar (Fig. 3). After all, despite its leaky nature, *stt7–9* cannot phosphorylate the LHCII protein LHCBM5 in state II conditions^25^ and cannot perform Stt7-mediated qT^13^,^29^. The differences in the behaviour of *stt7-7* and *stt7–9* during the quenching analysis are therefore only apparent under actinic light. Stromal residues of the LHCSR3 N-terminal (Ser-26, Ser-28, Thr-32, Thr-33, and Thr-39), can be phosphorylated in wild-type and *stt7–9*, but not in a non-leaky Stt7-deficient mutant^25^. We suggest that Stt7 phosphorylation of LHCSR3 is involved in the LHCSR3-mediated and light-dependent qT, which could explain the smaller increase in *F*_m_’ early during the quenching analysis by *stt7-7* compared to *stt7–9*. It is tempting to speculate that such LHCSR3 phosphorylations may also explain the deficiency of qE in *stt7-7*. However, a difference in the phosophorylation level of LHCB4, or other proteins that occur in *stt7–9*, compared with a non-leaky Stt7 mutant^25^, may also be responsible.

In summary, the rapid and slower decreases of *F*_m_° during a transition to state II (Fig. 3 inset) kinetically mirrored the rapid and slower increases in *F*_m_’ during transition to state I (Fig. 4c). With the use of *npq4, stt7-7* and *stt7–9* we are able to deduce that LHCSR3 is required for the more rapid transitions of qT, while only Stt7 kinase activity was involved in the slower transitions. Furthermore, Stt7-mediated phosphorylations are also involved in qE.

Previously, it has been shown that STN7 kinase of Arabidopsis has H_2_O_2_-sensitive exposed thiol groups^20^ and that LHC phosphorylations mediated by Stt7 kinase in *C. reinhardtii* were inhibited by H_2_O_2_^21^. It is now apparent that the more rapid transition to state II, which involves LHCSR3, is more sensitive to H_2_O_2_ than the slower transition to state II, which involves only Stt7 kinase (Fig. 3). Furthermore, the involvement of LHCSR3 explains why measurements of *npq4* were much less influenced by H_2_O_2_ (Fig. 3). An explanation to why LHCSR3-mediated qT is particularly sensitive to H_2_O_2_ could be that a smaller change in phosphorylation levels leads to a larger level of regulation than LHCII phosphorylation, which merits further investigation. A transition in the reverse direction was also sensitive to H_2_O_2_, as shown by the delayed transition to state I in wild-type cells treated with catalase. This phenomenon can be explained by H_2_O_2_ slowing Stt7 kinase activity, which accelerates the transition to state I^21^. As this effect was seen after removing H_2_O_2_ rather than by its addition, this level of regulation is clearly operational under standard lab conditions and with physiological levels of H_2_O_2_. Transitioning to state I during a sudden increase in light intensity has been previously described as a mechanism that facilitates qE induction by increasing light absorption by PSII^13^. Here we showed that catalase delayed the onset of qE by approximately 2.5 min in wild-type (Fig. 6) and *stt7–9* (Supplementary Fig. 4), both of which can phosphorylate LHCSR3. In conclusion, H_2_O_2_ production in the chloroplast can benefit *C. reinhardtii* by adjusting to a rapid increase in light intensity through a process involving Stt7 and LHCSR3, and potentially Stt7-mediated phosphorylation of LHCSR3.

## Materials and Methods

### Strains and Growth Conditions and high light pre-treatments

*Chlamydomonas reinhardii* wild type (wild-type) strain T222 (in the 137C background), *stt7–9* (a leaky mutant with an estimated 6-fold decrease in Stt7 kinase activity^25^) and *stt7-7* (a totally Stt7-deficient mutant, J-D. Rochaix, personal communication) were gifts from J-D. Rochaix, University of Geneva. *npq4*^11^ (CC-4614) was purchased from the Chlamydomonas Centre (www.chlamycollection.org). Cultures were initiated in Tris-Acetate-Phosphate media (TAP)^30^, adjusted to pH 7.0, and grown mixotrophically under low light (50 μmol quanta m^−2^s^−1^). To transfer cells to photoautotrophic conditions TAP cultures were pelleted for 2 min at 1600 *g* and suspended in Tris-HCl-Phosphate media (THP; identical to TAP except the pH was adjusted to 7.0 with HCl rather than acetic acid) and cultivated under low light while being bubbled with sterile air, achieved with a 0.22 μM air-filter. Cells were in THP for at least 24 h before experiments began, which is well beyond the time for residual acetate to be consumed that can affect photosynthetic performance and ROS production^31^. Liquid cultures were rotated at 80 rpm at 20 °C and kept in the exponential growth phase below 5 × 10^6^ cells ml^−1^ by regular dilution. Adjustment to 10 μg chlorophyll ml^−1^ was made immediately before pre-high light treatments.

High light was provided by a 250 W compact fluorescent lamp and cultures were kept between 20–25 °C with fan-assisted cooling. The high light intensity measured at the top and bottom of the culture was 300 and 200 μmol quanta m^−2^ s^−1^, respectively, and unless stated otherwise cultures were pre-high light-treated for 2 h.

### Photosynthetic pigments

Carotenoids were measured from 10 mg of lyophilized cells extracted in 1 mL of ice-cold acetone by shaking (TissueLyser II, Qiagen, Düsseldorf, Germany) at 30 Hz for 2 min with two 2 mm glass beads before centrifugation at 26,000 g for 45 min. Ten μl of the supernatant was injected using an Agilent 1100 HPLC system equipped with a LiChrospher 100 RP-18 (5 μm) column (Agilent Technologies, Santa Clara, California, USA). Peak identity and quantification was made against individual standards with absorbance at 440 nm. Total chlorophyll and chlorophyll *a* and *b* were measured according to^32^ in 80% acetone.

### Photosynthetic Measurements

Pulse amplitude-modulated (PAM) chlorophyll fluorescence measurements were made with a PAM 2500 (Walz GmbH, Effeltrich, Germany). Maximum chlorophyll fluorescence (*F*_m_) was measured with a 200 ms saturating pulse. Cultures of 1.5 mL were constantly stirred with a magnetic bar during measurements. For measuring the light dependency of qE induction cells were first allowed to recover from high light for at least 1 h and then treated with far-red light to fully achieve maximum *F*_m_ (*F*_m_^o^) Increasing intensities of light were provided for 1 min intervals after which *F*_m_’ (*F*_m_ under actinic light) was measured in order to calculate qE via (*F*_m_^o^ − *F*_m_’)/*F*_m_’. For measuring the state II to I transition, cells were treated with far-red light to achieve state I, as observed when *F*_m_^o^ no longer increased (typically 10 min when saturating pulses were kept 90 s apart). Induction of state II was measured by following the decrease in *F*_m_ after the far-red light was switched off. H_2_O_2_ was added 1 minute before measurements from a stock of 100 mM. For splitting the qE and qT responses cells were dark-adapted for various times, as stated in the Figure legend, before treating at a light intensity of 474 μmol quanta m^−2^ s^−1^. The *F*_m_’ was measured every 30 s. To observe the effects of nigericin the cell wall-less wild-type strain (cw15) was used, because nigericin can enter its cells and the strain possesses typical LHC phosphorylation patterns and can accumulate LHCSR3 (Roach, unpublished). Nigericin was used at 10 μM from a 10 mM stock dissolved in methanol. To assess PSII antenna size via *F*_m_^o^ cells were first treated for 1 min in the dark with 10 μM 3-(3,4-dichlorophenyl)-1,1-dimethylurea (DCMU) from a 10 mM stock dissolved in methanol.

Chlorophyll fluorescence emission between 650–750 nm was measured at 77 K with a Cary Eclipse fluorescence spectrometer (Varian, Mulgrave, Australia) at an excitation of 440 nm (5 nm slit width) in samples capillary-loaded in glass Pasteur pipettes and immediately frozen in N_2(l)_ before measurement.

### Analysis of LHCSR3, PsbA and threo-phosphorylated protein levels

Total cellular proteins were extracted in 2% SDS in 100 mM TRIS-HCl, pH 6.8, containing a protease inhibitor cocktail (Complete Mini, Roche Diagnostics, Switzerland). Proteins were quantified using the bicinchoninic acid assay (Sigma-Aldrich, St Louis MO, USA), loaded at 20 μg protein/sample and separated by PAGE using 12% acrylamide gels at 40 mA for 1.5 h. For western blotting separated proteins were transferred to nitrocellulose membranes at 40 mA/gel for 1 h, which were subsequently blocked in 5% fat-free milk powder before incubating with the LHCSR3 (Agrisera, Sweden) or anti-phospho-threonine antibody (Cell Signalling Technologies, USA) at 1:10,000 dilution or PsbA antibody (Agrisera, Sweden) at 1:25,000 dilution. The peroxidase-coupled antibodies were visualised with enhanced chemiluminescence (Amersham, GE Healthcare, UK) and light sensitive film (Amersham, GE Healthcare, UK).

### Statistics

Significant differences at *P* < 0.05 were calculated using IBM SPSS (v.21) and one-way ANOVA with Tukey’s post-hoc test.

## Additional Information

**How to cite this article**: Roach, T. and Na, C. S. LHCSR3 affects de-coupling and re-coupling of LHCII to PSII during state transitions in *Chlamydomonas reinhardtii. Sci. Rep.* **7**, 43145; doi: 10.1038/srep43145 (2017).

**Publisher's note:** Springer Nature remains neutral with regard to jurisdictional claims in published maps and institutional affiliations.

## Supplementary Material

Supplementary Figures

## Figures and Tables

**Table 1:** The differences in pigment composition and chlorophyll fluorescence parameters of wild-type and *npq4* cells after the high light pre-treatment.

	WT	SD	*npq4*	SD	% from WT
Lutein	15.06	1.32	16.03	0.89	+6.4
Total VAZ	9.09	0.43	10.31	0.77	+13.4*
Chl. *a:b*	2.49	0.11	2.70	0.08	+8.5*
*F*_v_/*F*_m_	0.63	0.01	0.61	0.01	−3.8*
*F*o (DCMU)	0.100	0.004	0.107	0.003	+7.0

‘WT’ = wild-type, ‘Total VAZ’ = total xanthophyll pool of violaxanthin, antheraxanthin and zeaxanthin calculated on a mol basis and expressed as molx100:mol total chlorophyll, as for lutein. ‘Chl. *a:b*’ = ratio of chlorophyll *a:b* c*a*lculated on a mol basis, ‘*F*_v_/*F*_m_’ = maximum quantum yield of PSII, ‘*F*o (DCMU)’ = Fo measured in cultures at 10 μg mL^−1^ chlorophyll in the presence of 10 μM DCMU, ‘% from WT’ was calculated as (*npq4-*WT)/WT × 100 and ‘*’ corresponds to a significant difference (*P* < 0.05), n = 4 ± SD.
