# Supplementary material for: LHCSR3 affects de-coupling and re-coupling of LHCII to PSII during state transitions in Chlamydomonas reinhardtii
Source: Sci Rep. 2017 Feb 24;7:43145. doi: 10.1038/srep43145 (PMC5324048; doi:10.1038/srep43145)
Supplement: Supplementary Figures [file srep43145-s1.pdf]

Supplementary information for the paper titled:

LHCSR3 affects de-coupling and re-coupling of LHCII to PSII during state transitions in *Chlamydomonas reinhardtii*

Thomas Roach[1], Chae Sun Na[1,2]
[1]Institute of Botany, Leopold-Franzens-Universität-Innsbruck, Sternwartestraße 15, 6020 Innsbruck, Austria
[2]Institute of Ecological Phytochemistry, Department of Plant Life and Environmental Science, Hankyong National University, Anseong 456-749, Gyeonggi-do, Republic of Korea

For correspondence: Thomas Roach (thomas.roach@uibk.ac.at)

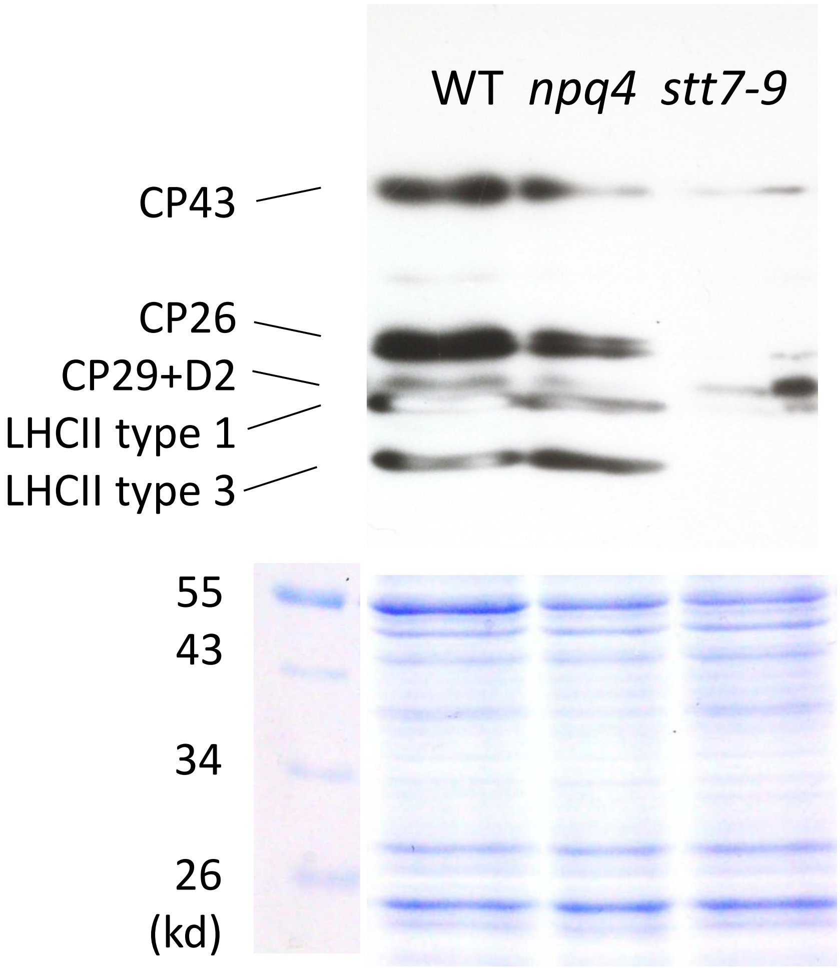

Supplementary Figure 1. The threo-phosophorylation pattern of thylakoid proteins in 10 min dark-adapted wild-type (WT), *npq4* or *stt7-9* cells. A parallel ran Coomassie stained gel showing the same molecular weight proteins of the western blot is shown as a loading control.

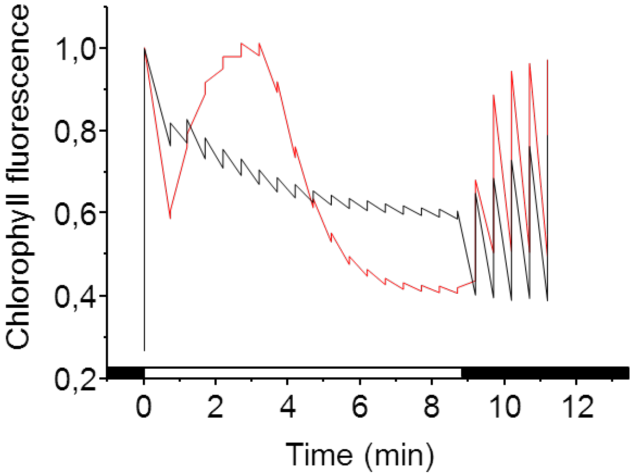

Supplementary Figure 2. Chlorophyll fluorescence differences of cells acclimated to either high or low light (250 or 50 µmol quanta m^{-2} s^{-1}, respectively) for 16 h, as represented by the red or black trace, respectively. Fluorescence before a saturating pulse and F_m are indicated every 0.5 min by the lower and upper limits, respectively, of each vertical line of each trace. Cells were dark-adapted for 5 min before measurements. Actinic illumination (474 µmol quanta m^{-2} s^{-1}) and dark are represented by the white and black bars on the X-axis, respectively. Saturating pulses to measure F_m were made every 0.5 min. Data is normalised to the F_m° value at time 0.

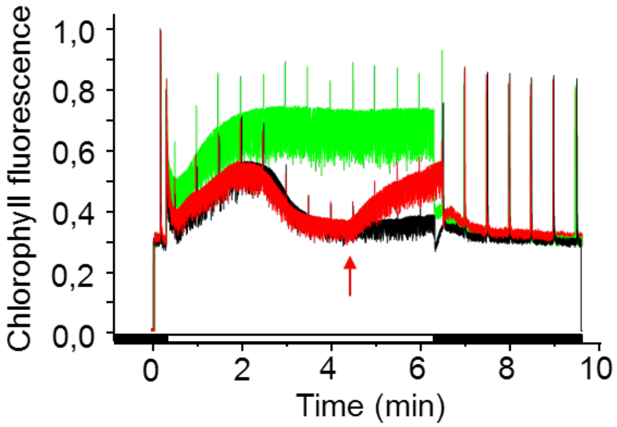

Supplementary Figure 3. The effect of 10 µM of the ΔpH dissipater nigericin of chlorophyll fluorescence traces, either added 5 min before light treatment (green trace), at the time indicated by arrow (red trace), or not at all (black trace). Cells were dark-adapted for 5 min before measurements. Actinic illumination (474 µmol quanta m^{-2} s^{-1}) and dark are represented by the white and black bars on the X-axis, respectively. Saturating pulses to measure F_m were made every 0.5 min. Data is normalised to the F_m° value at time 0.

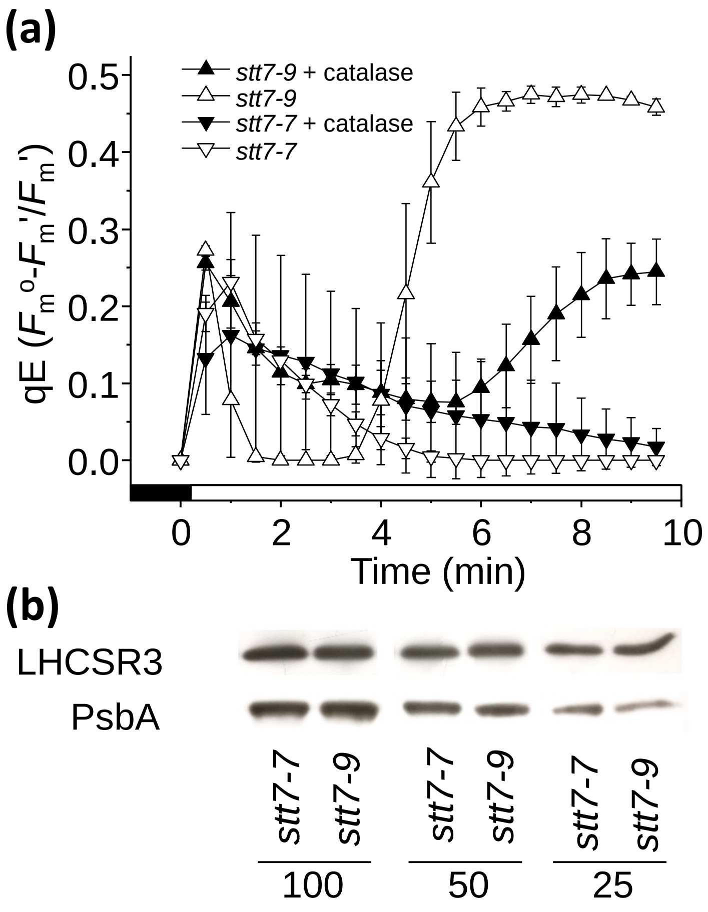

Supplementary Figure 4. A comparison of qE between the "non-leaky" mutant *stt7-7* with the "leaky" mutant *stt7-9* and the effect of catalase. **(a)** *stt7-9* (upward triangles) and *stt7-7* (downward triangles) cells were treated for 4 h with high light, then dark adapted for 20 min before a quenching analysis at 474 µmol quanta m^{-2} s^{-1}, as indicated by the white bar on the X-axis, in the presence (filled symbols) or absence (open symbols) of 500 U mL^{-1} catalase, n=3±SD. **(b)** The protein levels of LHCSR3 of cells after the high light treatment. Each lane contained 1, 0.5 and 0.5 µg total chlorophyll in lanes labelled 100, 50 and 25, respectively. The PsbA protein level is shown as a loading control.

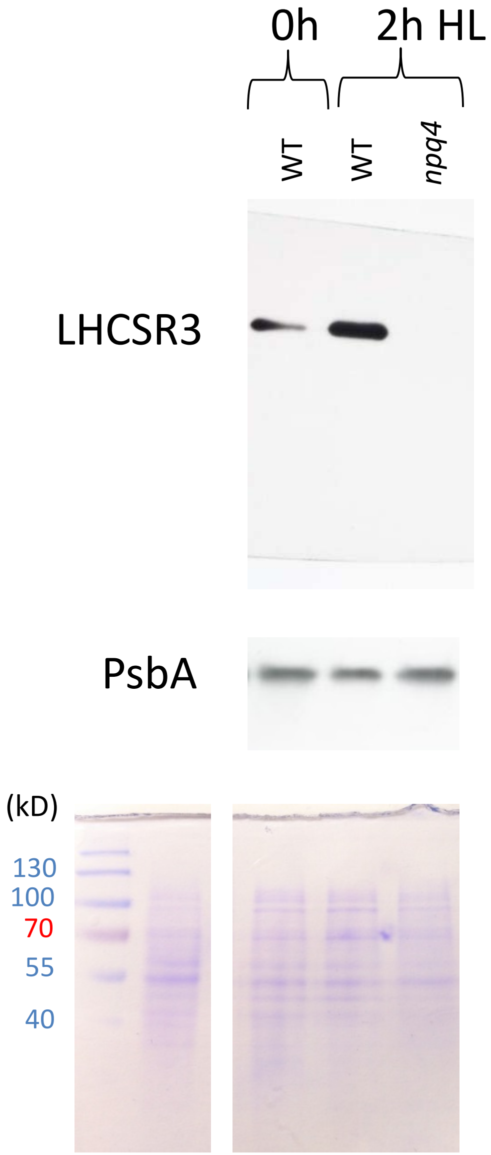

Supplementary Figure 5. The full-length blot of LHCSR3 of wild-type (WT) after 0 and 2 h high light (HL) and *npq4* after 2 h HL. The amount of PSII reaction centre (PsbA) and a parallel ran Coomassie stained gel with molecular markers are shown as loading controls.

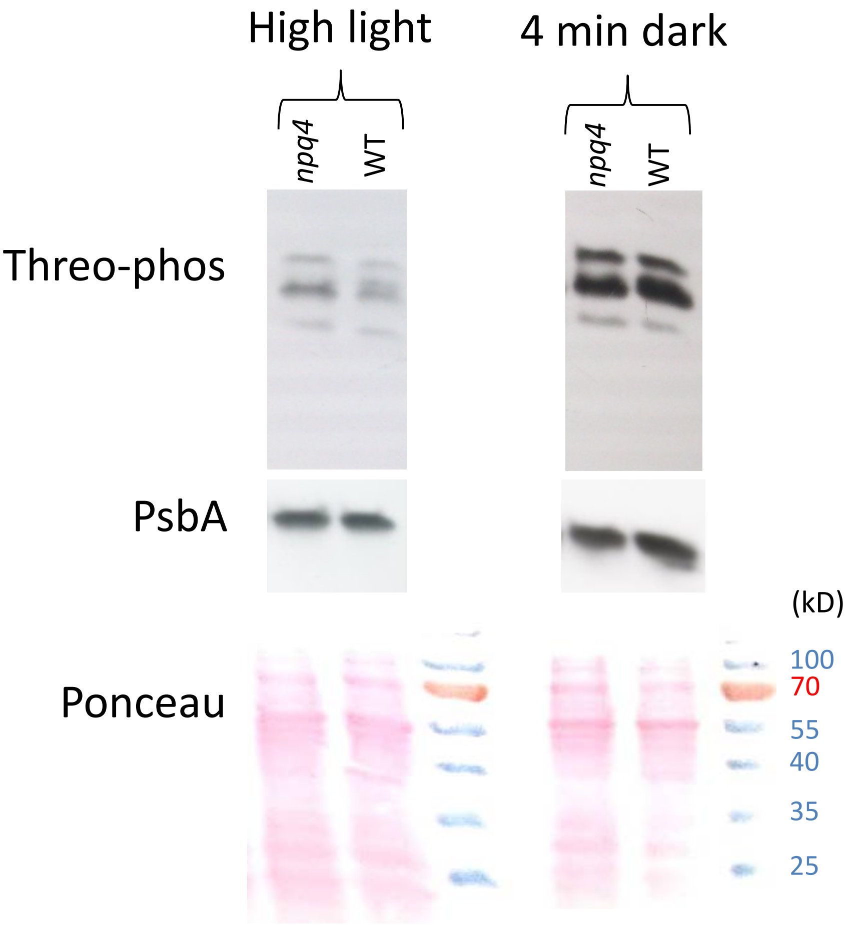

Supplementary Figure 6. The full-length blot of threo-phosophorylation patterns of thylakoid proteins of wild-type (WT) and *npq4* in high light and after 4 min dark. The amount of PSII reaction centre (PsbA) and Ponceau staining of the nitrocellulose membrane are shown as loading controls.
